# Evaluating Fitness in Older Acute Myeloid Leukemia Patients: Balancing Therapy and Treatment Risks

**DOI:** 10.3390/jcm13216399

**Published:** 2024-10-25

**Authors:** Matteo Molica, Martina Canichella, Elias Jabbour, Felicetto Ferrara

**Affiliations:** 1 Department of Hematology-Oncology, Azienda Universitaria Ospedaliera Renato Dulbecco, 88100 Catanzaro, Italy; 2 Hematology Unit, St. Eugenio Hospital, 00144 Rome, Italy; martina.canichella@aslroma2.it; 3 Department of Leukemia, MD Anderson Cancer Center, The University of Texas, Houston, TX 77030, USA; ejabbour@mdanderson.org; 4 Division of Hematology, Cardarelli Hospital, 80131 Naples, Italy; ferrarafelicetto@gmail.com

**Keywords:** fitness, acute myeloid leukemia, dynamic fitness

## Abstract

Assessing the suitability of older adults with acute myeloid leukemia (AML) for intensive chemotherapy or stem cell transplantation remains a long-standing challenge. Geriatric assessment, which involves the evaluation of multiple dimensions of health, may influence a patient’s ability to tolerate intensive or mild-intensity approaches, including treatment-related mortality. Prospective studies are required to validate different fitness criteria, in addition to making it possible to compare the effectiveness of geriatric assessment-based fitness against other criteria, in order to identify which aspects of geriatric assessment are linked to treatment tolerance. It is hoped that validation studies will include different groups of patients receiving either intensive or lower-intensity chemotherapy. At a minimum, geriatric assessment should involve the measurement of the comorbidity burden, cognition, physical function, and emotional health—factors previously associated with mortality in AML. These assessments should be conducted before starting chemotherapy in order to minimize the treatment’s impact on the results. While treatment tolerance has traditionally been evaluated through toxicity rates in solid tumor patients, AML treatment often results in high toxicity rates regardless of the intensity. Therefore, early mortality should be the primary endpoint for assessing treatment tolerance, given its significant and clear implications. Other important endpoints might include declines in functional status and quality of life and treatment adjustments or discontinuation due to toxicity. Validating these fitness criteria is essential for guiding treatment choices, improving supportive care, determining trial eligibility, interpreting study outcomes, and informing drug labeling.

## 1. Introduction

Acute myeloid leukemia (AML) is a very heterogeneous disease characterized by the uncontrolled proliferation of clonal hematopoietic cells in bone marrow, the blood, and extramedullary sites [1,2]. It is the most common type of acute leukemia in adults, presenting a median age at baseline of 68 years [3]. The prognosis is closely related to age, with progressive worsening in elderly patients [1,3]. Of note, in the age group with the highest incidence, 60–70 years, the prognosis is particularly poor, with individuals in this age range showing a 5-year overall survival (OS) rate of approximately 20%. Several factors account for the short survival; unfavorable genetic characteristics are more frequent at advanced ages, and there are often no treatment options for elderly patients other than supportive therapy. However, in the coming years, these data are expected to improve. The introduction of hypomethylating agents (HMAs) has drastically changed the treatment of patients who are not candidates for chemotherapy. Indeed, novel drugs, alone or in combination with HMAs, have been approved and have demonstrated high efficacy and favorable toxicity profiles. Consequently, there is growing interest in refining the treatment eligibility criteria. At present, some international guidelines and expert groups have recommended careful assessment of a patient’s fitness rather than their chronological age to develop appropriate treatment options [4,5].

In this review, we first provide a brief overview of the current non-intensive treatment options for patients with AML and then focus on the assessment of fitness by analyzing the criteria used for its evaluation.

## 2. Non-Intensive Therapy for Newly Diagnosed Patients

Before the introduction of HMAs, treatment strategies for AML were divided into intensive chemotherapy and supportive care, the latter specifically for elderly unfit patients. The advent of azacitidine (AZA) and decitabine (DEC) represented a turning point in the treatment of AML, introducing a less-intensive strategy category. Despite limited activity as single agents, HMAs represent a platform for low-intensity combinations [6,7]. Over the years, with advancements in the molecular background of AML, new targeted drugs with high efficacy and good safety profiles have been developed. Simultaneously, the assessment of the eligibility of elderly patients has been refined, taking into account age, comorbidities, and several multiparametric tools. Consequently, a greater number of elderly patients, previously considered unfit, have been treated. At present, for patients not eligible for intensive chemotherapy, treatment options involving the following inhibitors are available: B-cell lymphoma 2 (BCL-2) inhibitors, FMS-like tyrosine kinase 3 (FLT3) inhibitors, isocitrate dehydrogenase 1 and 2 (IDH1/2) inhibitors, and smoothened inhibitors (SMO). The BCL-2 inhibitor venetoclax has been tested in combination with HMAs or low-dose cytarabine (LDAC), demonstrating high efficacy and low toxicity [8]. In the phase III VIALE-A trial, combining AZA with venetoclax resulted in better responses and overall survival (OS) for patients over 75 years of age or with comorbidities, demonstrating a CR rate of 66.4% compared to 28.3% for AZA alone (*p* < 0.001). Moreover, the median OS was 14.7 months versus 9.7 months, with a hazard ratio of 0.66 (95% CI 0.52–0.85, *p* < 0.001) [9]. In the VIALE-C trial, venetoclax was administered in combination with low-dose ara-C (LDAC) and compared to LDAC alone. A post hoc analysis with an additional six months of follow-up revealed that the combination of venetoclax and LDAC offered a survival benefit, with OS times of 8.4 months for the combination group versus 4.1 months for the monotherapy group (HR 0.7, 95% CI 0.5–0.98, *p* = 0.04) [10]. The IDH1 inhibitor ivosidenib was tested in combination with AZA versus AZA alone in IDH1+ AML patients over of age 75 years or with comorbidities in the phase III AGILE study [11]. Event-free survival (EFS) and the median OS were higher in patients who received ivosidenib. However, a comparison between ivosidenib in combination with AZA and HMAs in combination with venetoclax is not currently available. Thus, in elderly patients with IDH1 mutation, the question of whether to combine AZA with either venetoclax or ivosidenib currently remains unanswered, especially given that ivosidenib could be used as a salvage treatment in cases of relapse. The use of the IDH2 inhibitor enasidenib in combination with azacitidine is not currently approved; however, it was explored in the phase II AG221-AML-005 randomized study and showed promising results [12]. More recently, enasidenib as monotherapy demonstrated efficacy in elderly patients in inducing CR and the combination with AZA resulted in efficacy for those patients who did not respond to enasidenib alone [13]. Despite the success of gilteritinib in relapsed/refractory (R/R) FLT3+ AML in the ADMIRAL trial, in the first line, combination with AZA failed to demonstrate superior OS, as seen in the phase III LACEWING trial. However, in the first line, the combination of gilteritinib, AZA, and venetoclax demonstrated a high complete remission (CR) rate and a deep molecular response, which could be translated as improving outcomes [14]. Lastly, in the phase II BRIGHT AML 1003 trial, glasdegib and LDAC compared to LDAC alone showed a better OS in patients not eligible for intensive chemotherapy (with a median OS of 8.3 months versus 4.3 months) [15].

These strategies have revolutionized the therapeutic options available to elderly patients and ultimately improved their survival. It should also be considered that, for high-risk patients, these therapeutic options could represent a bridge to allogeneic stem cell transplantation (allo-SCT). In recent years, the eligibility criteria for allo-SCT have also undergone radical change, no longer considering only chronological age as an absolute parameter for access to this treatment. Indeed, with the introduction of reducing intensity conditioning (RIC), the improvement of prophylaxis for Graft-versus-Host Disease (GvHD), and supportive care, a greater number of elderly patients have undergone this potentially curative therapy.

## 3. Definition of Fitness

The aim of assessing the fitness of older patients with AML is to determine whether they are candidates for curative therapy that can achieve a durable CR. This thorough evaluation is intended to rule out treatments that might (1) exacerbate age-related frailties, (2) induce organ damage due to previous or current comorbidities, or (3) be difficult for the patient to adhere to because of their individual characteristics, in order to reduce the treatment-related mortality. Such risks could compromise the short-term life expectancy more than the disease (AML) itself, thereby supporting the decision to opt for more conservative treatments, such as non-intensive or supportive care. Although there are a larger number of scoring systems designed to assess the suitability for intensive chemotherapy, a universally accepted procedure for defining fitness is still lacking. In this context of ongoing uncertainty, factors related to the patient (such as performance status and age) and the disease (such as cytogenetics and blood count) continue to be integral to these systems, helping to distinguish patients who might benefit from an intensive approach from those who might not.

## 4. Determination of Ongoing Criteria to Evaluate Fitness

### 4.1. Age and Comorbidities

Traditionally, eligibility for intensive chemotherapy was thought to be primarily contingent on an individual’s age. Age-related influences on cases of AML and disease-related characteristics are well-established; these effects lead to an increased risk of early death following induction chemotherapy in addition to a decreased likelihood of full response and long-term survival [16]. The World Health Organization (WHO) designates 65 years old as the age at which a patient becomes “elderly”. Given the shorter life expectancy of individuals living in underdeveloped countries, the United Nations deems the transition to occur at 60 years of age. However, age alone cannot be considered as an absolute factor for identifying patients classified as ineligible for intensive treatment, even if fitness and age are often largely correlated. In daily practice, there are cases where a younger patient with comorbidities performs worse than an older patient without comorbidities. The finding that chronological age should not be the primary criterion used to establish fitness or treatment selection begs the important question of whether biological age and chronological age actually correlate [17]. In a retrospective cohort analysis, clinical practices associated with AML therapy and outcomes were examined in AML patients who were at least 66 years of age. Data from Medicare enrollment and claims files, in addition to the Surveillance, Epidemiology, and End Results (SEER) program database, were used in the analysis. In total, 40% of the 8336 eligible patients underwent AML treatment within three months of diagnosis [18]. It was also found that treatment rates rose from 35% in 2000 to 50% in 2009. Compared to patients who were not receiving therapy, those undergoing treatment had a decreased prevalence of secondary AML, comorbidities, and poor performance indicators. Treatment lowered the risk of death during the observation period by 33%; patients receiving intensive therapy had a longer median OS (18.9 months) than those receiving HMAs (6.6 months) or receiving no treatment (1.5 months); patients under 75 years of age had a similar mortality risk reduction to those over 75 years of age. Comorbidities, low-performance markers, and previous myelodysplastic syndrome (MDS) were all linked to early mortality. Conversely, a sizable (*n* = 980), retrospective, and single-center study involving AML patients who were diagnosed between 1995 and 2016 and who were at least 70 years of age revealed that the use of HMAs significantly improved survival (median OS = 14.4 months) when compared to supportive care (2.1 months), low-intensity therapy (5.9 months), and high-intensity therapy (10.8 months). In this study, 43% and 57% of patients suffered from de novo AML and secondary AML, respectively [19,20]. Of the patients, 37% received high-intensity therapy, 26% received HMAs, 9% received low-intensity therapy, and 28% received supportive care. Clinical factors that have been found to affect OS include age, white blood cell count, platelet count, hemoglobin level at diagnosis, poor-risk cytogenetics, PS, front-line therapy, and secondary AML. These findings highlight the advantages of AML therapy and indicate some important variables to take into account when assessing older patients’ fitness. More recently, Lazarevic et al. used information from the Swedish AML registry to present clinical and diagnostic aspects focusing on patients who were 80 years of age or older. Complex and monosomic karyotypes were more prevalent in this group of patients, despite the fact that the older patients in this study tended to undergo less morphologic sub-classification and genetic screening. The prevalence of secondary AML was lowest in patients under the age of 85 but highest in those between the ages of 70 and 80 [21]. Moreover, in the subgroup of older patients, there is an enrichment of the TP53 gene mutation, which is associated with well-known chemoresistance [22].

These findings point to slight variations in clinical AML subgroups between the ages of 70 and 100 years, and they support the gathering of molecular information on these patients, especially in light of the development of novel treatments, many of which may be advantageous to individuals with particular AML subtypes (such as secondary AML) or molecular characteristics.

The potential for comorbidities to impact toxicity and treatment response has been noted. In order to determine the overall suitability of a specific treatment, comorbidity assessment is helpful [23]. Etienne et al. identified comorbidities as an independent predictor of CR after induction therapy [24]. The Charlson Comorbidity Index (CCI) and the Hematopoietic Cell Transplantation (HCT)–Specific Comorbidity Index (HCT-CI) have been historically validated in order to identify several potential outcomes in AML [25]. The HCT-CI takes into account objective criteria to identify comorbidities and not only summarizes the number of conditions but also determines their burden [26]. Comorbidities with the highest scores (3 points) in the HCT-CI are pulmonary disease, hepatic abnormalities, heart valve disease (except mitral valve prolapse), and a prior solid tumor. Retrospectively analyzing 177 AML patients aged > 60 years who received intensive chemotherapy, it was found that those with an HCT-CI score ≥ 3 showed an early mortality rate of 29% vs. 3% and 11% in patients with scores of 0 and 1–2, respectively (*p* < 0.001). In view of these findings, intensive therapy may be appropriate for a patient who has well-managed comorbidities. Assessment of the comorbidities may help to determine a patient’s general suitability for intensive therapy; however, it cannot provide a 100% guarantee of the acceptability of AML treatment because other aspects need to be taken into account.

### 4.2. Performance Status

Regardless of age, oncology performance status (PS) measures such as ECOG PS or Karnofsky PS (KPS) can help to identify AML patients at a higher risk of early death or treatment-related mortality following intensive chemotherapy. According to Kadia et al., poor PS appears to be significantly more adversely relevant to increasing age [27].

A number of researchers have explored potential treatment approaches in patients with poor PS. The authors of a study including 2767 AML patients belonging to the Swedish Acute Leukemia Registry assessed the influence of the decision to treat on outcomes [3]. The percentage of patients undergoing intensive therapy decreased as the PS worsened. Age and PS both affected the thirty-day mortality rates; however, older patients with better PS had reduced early death rates, whereas patients with low PS had higher early death rates at all ages. Early death was reported in 36% of patients with a PS of 3–6 who had received intensive therapy compared to 52% of patients who had received the best available supportive care (*p* = 0.023). Intensive therapy may be beneficial for certain patients, as there were some long-term survivors among patients with compromised PS, despite the fact that the early mortality rate was increased in all age categories.

The results of these trials indicate that most older AML patients would benefit from treatment and that intensive approaches are most effective compared to low-intensity therapy (azacitidine or decitabine) and supportive care alone. Therefore, while PS and age are strongly correlated, they are not adequate on their own to determine fitness. In older AML patients, varying degrees of comorbidity—some of which may be best managed—highlight the need for improved methods of determining fitness. Therefore, more detailed methods are required to more accurately select patients for intensive therapy.

### 4.3. Multi-Parameter Assessment Tools

The use of geriatric assessment tools and multi-parameter assessments has been considered to provide additional prognostic information to define fitness in AML patients. Although there is currently no agreement on which health domains to include and how to best incorporate various factors, geriatric assessment tools evaluate multiple health domains to more comprehensively assess patient fitness. They may also help to improve risk stratification and personalize therapy for older AML patients.

The Geriatric Assessment in Hematology (GAH) was proposed as a tool for the rapid assessment of elderly patients affected by hematologic malignancies and comprises eight dimensions of performance, mental status, and health status that provide a score of 0–8. The tool was validated after an analysis of 349 patients aged ≥ 65 years with hematologic malignancies, including AML [28]. The GAH score corresponded with the ECOG PS and KPS, when excluding the comorbidities domain. Higher GAH score groups of ≤1, 2–6, and >6 appeared predictive of survival (*p* < 0.001).

In patients with newly diagnosed AML who were at least 60 years of age and receiving intensive therapy, the authors of a prospective cohort study assessed the predictive value of geriatric assessments for OS, which included measures of cognitive function, depressive symptoms, distress, physical function, and clinical characteristics [29]. Age and ECOG PS were not related to OS; instead, they were related to the cytogenetic risk group, previous MDS, and baseline hemoglobin level. Low physical performance (Short Physical Performance Battery score < 9) and poor cognitive function (Modified Mini-Mental State score < 77) among geriatric assessment measures were linked to poor OS and boosted the prediction efficacy of the more common clinical indicators by 60%. The authors of another study investigated quality-of-life and geriatric evaluations in 195 individuals with AML and MDS who were older than 60 years of age [30]. The PS, activities of daily living (ADLs), comorbidities, and illness features were among the patient-related parameters assessed in the study. The European Organization for Research and Treatment of Cancer Quality of Life Questionnaire C30 (QLQ-C30) fatigue score of 50 and signs of dependence (ADLs < 100 and KPS < 80) offered the strongest prognostic information in the final model, excluding the known disease-related factors of poor-risk cytogenetics and bone marrow blasts.

Another study not only showed that geriatric assessments can predict treatment-related toxicities (i.e., grade 3 and 4 infections and renal impairment) but also found that reduced physical function, measured by the Short Physical Performance Battery (SPPB), and depressive symptoms, assessed by the Geriatric Depression Scale, were linked to lower survival rates. The study validated the relationship between the pre-treatment physical performance, specifically using the SPPB, and survival in older patients receiving intensive treatment. Notably, the researchers demonstrated that assessing the gait speed alone, a practical clinical test, offered comparable predictive value [31]. Furthermore, Min et al. analyzed 105 AML patients with geriatric assessment prior to intensive chemotherapy consisting of cytarabine and idarubicin. The evaluation included assessments of physical function, cognition, depression, distress, nutrition, and social support. Impairment in physical function measured by SPPB was associated with grade 3 to 4 infections (*p* = 0.024) and renal failure (*p* = 0.013) [32].

Incorporating geriatric assessments to profile patients, along with genetic profiling of leukemic cells, offers a cutting-edge precision medicine strategy to tailor treatment choices for older adults with AML. In a recent study, which evaluated patients using geriatric assessment and the genetic profile, the mortality at 30 days from diagnosis was 6.8% and at 90 days was 21.9%. This mortality rate compared favorably to an unmatched historical cohort of patients ≥ 60 years treated previously at the same Center, where the 30-day mortality was 30%, and the 90-day was 41% [33].

A prognostic model was developed in the study by Kantarjian et al. [34] to predict outcomes in older AML patients by classifying patients into risk groups based on a variety of patient- and disease-related characteristics. In comparison with the unfavorable-risk group, the OS and CR rates in the favorable-risk and intermediate-risk groups were higher. The predictive significance of the prognostic variables, such as the mutational status, on the clinical outcomes was examined in a prospective trial involving 909 AML patients who were over 60 years of age [35]. Age, karyotype, NPM1 mutation status, white blood cell count, LDH level, and CD34 expression were found to be independent prognostic predictors of OS through multivariate analysis; these variables were then given relative point values. Four prognostic profiles—favorable risk cytogenetics, intermediate risk cytogenetics with favorable risk features (score ≤ 3), intermediate risk cytogenetics with adverse risk features (score > 3), and high-risk cytogenetics—were identified based on the total points and a patient’s cytogenetic risk. In these groups, the patients’ respective OS rates were 40%, 30%, 11%, and 3%.

It is possible that cytogenetic analysis results indicating risk may not be readily accessible to AML patients who need to start therapy immediately. Thus, with or without knowledge of cytogenetic and molecular risk, the risk scores were determined using standard clinical and laboratory factors, such as body temperature, age, hematologic measurements, LDH level, and AML subtype, with the use of a web-based program [36]. These factors may help in treating these individuals, as they were found to be independently and strongly correlated with CR and early mortality (Table 1).

## 5. Fitness Criteria

Ferrara and colleagues introduced for the first time a definition of “unfitness” for both intensive and non-intensive chemotherapy in patients with AML. This definition was formulated through a Delphi consensus-based approach, which involved the Italian Society of Hematology (SIE), the Italian Society of Experimental Hematology (SIES), and the Italian Group for Bone Marrow Transplantation (GITMO) [5]. The process emphasized the importance of avoiding therapies that could exacerbate age-related frailty, cause organ intolerance, or shorten life expectancy due to existing comorbidities. These criteria specify that unfitness for intensive chemotherapy requires meeting at least one of nine conditions, whereas unfitness for non-intensive chemotherapy requires fulfilling at least one of six conceptual criteria. Additionally, the panel identified 15 operational criteria to further define unfitness for both intensive and non-intensive chemotherapy. These consensus-based definitions link geriatric and comorbidity factors to specific treatment decisions for AML patients, offering widely applicable criteria that help predict the benefit of varying treatment intensities across different patient fitness levels (fit, unfit, and frail).

In their recent study, Borlenghi et al. retrospectively applied the Ferrara criteria to a large cohort of 699 consecutive AML patients treated across eight hematologic centers in order to validate their clinical influence on clinical practice [37]. The criteria were functional in 98% of the patients, and fitness was found to be an independent and significant predictor of survival, as confirmed by univariate and multivariate analysis results. The study authors stated that these straightforward criteria, when combined with biological risk assessment, could serve as an effective tool for tailoring the treatment intensity to individual AML patients. In a further study, the authors also explored the integration of European Leukemia Net (ELN) risk categories with the Ferrara criteria to identify subgroups with varying prognoses and to guide treatment decisions for patients with secondary AML, a diverse patient population. In a retrospective analysis of 280 consecutive secondary AML patients over the age of 64, diagnosed between 2008 and 2015, the median OS was 10.1 months for fit patients compared to 4.2 and 1.8 months for unfit and frail patients, respectively [38]. The results of this study demonstrate that fitness evaluation is a strong predictor of patient outcomes, leading the authors to recommend that, in addition to age, fitness assessment should be a standard practice for older AML patients.

In a retrospective analysis, Palmieri et al. applied the Ferrara criteria to 180 consecutive AML patients (125 over 60 years of age and 55 under 60 years of age, with a median age of 66) treated at a single institution [39]. The analysis results revealed that risk stratification did not vary between younger and older patients, indicating that risk is not solely determined by age. The study authors found a strong correlation between the operational criteria and patient outcomes, with OS rates of 15.3 months for fit patients, 8.6 months for unfit patients, and 1 month for frail patients. Subsequently, Palmieri et al. applied the Ferrara criteria to retrospectively assess the fitness of 655 adults who received intensive chemotherapy for AML at the Fred Hutchinson Cancer Research Center in Seattle [40]. This evaluation aimed to determine the accuracy of these criteria in predicting early mortality and survival. The findings showed that the criteria had good-to-very good accuracy in forecasting 28-day and 100-day mortality, outperforming the treatment-related mortality score. The accuracy improved further when combined with additional factors such as albumin levels or PS. The authors concluded that the Ferrara criteria, when used in combination with molecular and genetic data, can support informed decision making for AML patients, particularly those who are older or have comorbidities (Table 2).

In clinical practice, allogeneic hematopoietic stem cell transplantation (HSCT) is increasingly utilized, though it remains associated with significant morbidity and mortality, particularly in elderly patients. The European Society for Blood and Marrow Transplantation (EBMT) score considers factors such as age, disease status, time from diagnosis to transplant, and donor–recipient sex combination to assist in HSCT candidate selection [41]. In a 2021 study in Japan, the authors developed the NRM-J index, which incorporates age, sex, ECOG performance status, HCT-CI, and donor type to predict non-relapse mortality. This index proved to be significantly more accurate than the EBMT score in forecasting non-relapse mortality after HSCT, suggesting its potential value in treatment decision making [42].

## 6. The Concept of Clinical Dynamic Fitness

In elderly patients with AML, it is crucial to continuously reassess a patient’s clinical fitness during the course of treatment, especially when their clinical and biochemical parameters positively or negatively change. These changes often appear adverse, typically due to severe complications associated with the treatment itself, potentially causing infections or organ failure; however, improvements in the patient’s functional status can also occur, especially in those patients whose health condition is strongly affected by disease-related issues. For patients in whom significant changes occur during the course of treatment, reassessing clinical fitness at specific time points is necessary to tailor the type or intensity of therapy accordingly, ensuring that the treatment remains aligned with the patient’s current fitness level and the characteristics of their disease. An emblematic example of this situation is represented by the use of oral AZA; this small agent has been shown to improve survival when used as maintenance therapy in patients in CR after intensive induction and consolidation chemotherapy [43]. Such patients are considered eligible for HSCT according to the biological prognostic risk of their disease; however, they become unable to proceed because of a worsening condition related to intensive therapy or logistical reasons.

**Figure 1:**
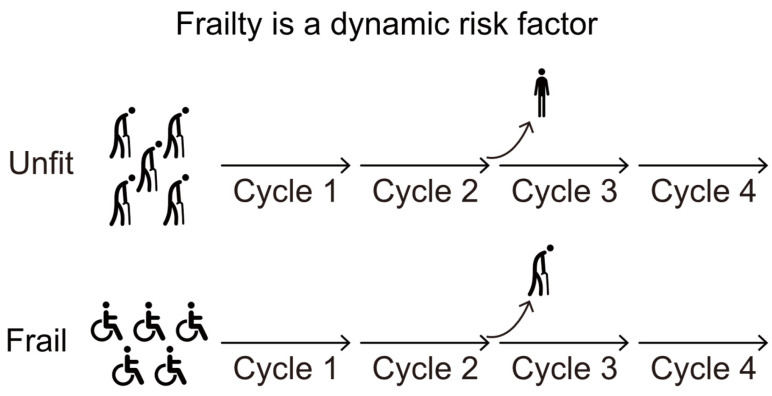
Visual representation of fitness improvement related to antileukemic therapy.

**Figure 2:**
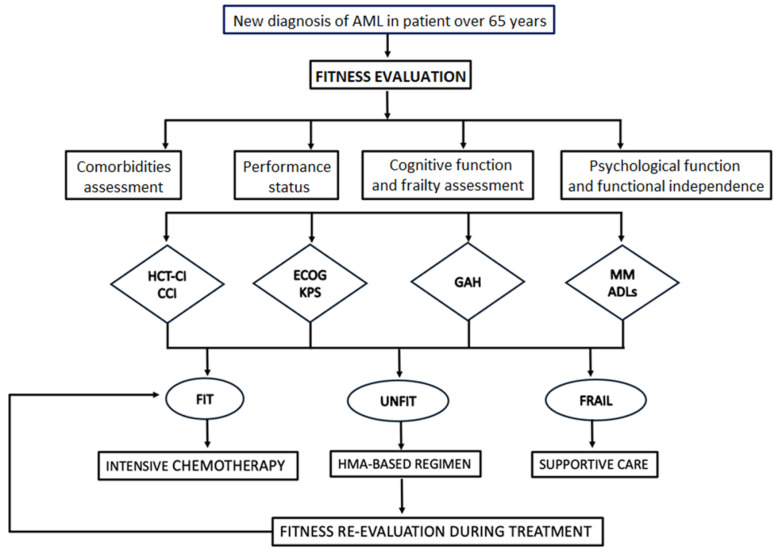
Flow-chart for assessing fitness evaluation in elderly patients at diagnosis and during treatment.

Conversely, during the course of lower-intensity treatment, patients initially considered unfit for intensive treatment may show significant improvements in their fitness condition, thereby becoming potential candidates to receive more intensive treatment. A post hoc analysis of two multicenter trials involving adults with newly diagnosed AML who were considered ineligible for intensive chemotherapy showed that approximately 10% of these patients eventually underwent allogeneic HCT after achieving CR [9,44]. These findings suggest that the initial fitness status in this study may have been significantly influenced by disease-related symptoms rather than pre-existing conditions unrelated to the disease. In such cases, reassessing the patient’s fitness could support a change in treatment strategy, potentially leading to better outcomes for some patients (Figure 1). We have provided a flowchart (Figure 2) useful for fitness assessment in the elderly patient both at diagnosis and during treatment.

## 7. The Concept of Biological Dynamic Fitness

Upon AML relapse or progression, it is not uncommon to identify leukemic clones harboring newly occurring mutations that were not detected at baseline. Therefore, during the course of treatments, the status of AML biology necessitates continuing assessments in order to monitor the potential evolution of the disease, either on its own or under the influence of AML therapies, which can determine crucial changes altering the clonal and sub-clonal pathways [45]. As targeted therapies gain increasing approval in AML therapeutic regimes, the ability to detect, through molecular retesting, an emerging leukemic clone at relapse or disease progression becomes a mandatory approach, not only for prognosis but also for guiding eventual salvage treatments.

As more targeted agents become available, the choice of the initial treatment may be based more on the characteristics of the disease than on the patient’s medical fitness. Emerging data suggest that certain lower-intensity therapies (such as VEN combined with an azanucleoside) could be as effective as intensive chemotherapy, while being better tolerated [46]. Looking forward, treatments tailored to the biological profile of AML rather than age or fitness could help minimize toxicity during remission induction, making it easier for patients to tolerate subsequent intensive therapies, including HSCT.

## 8. Conclusions

The range of frontline treatment options for AML has significantly broadened in recent years. Historically, treatment options were limited to a choice between intensive induction chemotherapy and non-intensive options such as supportive care, with patient fitness being the primary factor in deciding between these approaches. At present, treatments vary in intensity and toxicity and are tailored more specifically to the disease’s cytogenetic and mutational characteristics. Ongoing studies are eagerly anticipated, particularly those involving triplet therapy combining venetoclax/HMAs with targeted treatments. Moreover, new agents are being incorporated into initial treatment plans to achieve stronger responses. However, there is a notable lack of prospective studies comparing intensive regimens such as 7 + 3 or CPX-351 with venetoclax/AZA, which would clarify whether patients deemed fit for intensive chemotherapy might actually benefit more from the less intensive options now available. Tools such as Ferrara et al.’s consensus criteria [5] can help determine unfitness for intensive chemotherapy; moving forward, however, it will be important to increasingly integrate functional and genomic biomarkers to guide treatment decisions, given the expanding array of effective therapies.

## Figures and Tables

**Table 1:** Different criteria and scores to evaluate patients’ fitness.

Criteria	Tools	Findings
Age and comorbidities	CCI HCT-CI	Although fitness is largely associated with age and the WHO has defined the chronological age of 65 years as the threshold for being considered elderly, chronological age alone should not be the only criterion for treatment decisions. On the other hand, the presence of comorbidities is crucial in refining both the response to treatment and the assessment of toxicity. However, the management of comorbidities should not rule out the possibility of intensive treatment [3,17,20,21].
Performance status	ECOG KPS	Performance status is an age-related but also age-independent tool that helps identify patients who are unfit for intensive chemotherapy. Additionally, the PS is correlated with both treatment response and overall survival [27].
Multi-parameter assessment tools	GAH SPPB MMS ADLs	The evaluation of geriatric assessment using various tools is useful in defining fitness in elderly patients. However, several studies demonstrated that a more accurate determination of fitness needed the integration of multiple geriatric assessment tools with the clinical and biological characteristics of the disease [27,28,29,31,34,35].

CCI: Charlson Comorbidity Index; HCT-CI: Hematopoietic Cell Transplantation-Specific Comorbidity Index; KPS: Karnofsky PS; GAH: Geriatric Assessment in Hematology; SPPB: Short Physical Performance Battery; ADLs: activities of daily living.

**Table 2:** Ferrara criteria and subsequent validation studies.

Criteria	Methodology	Findings
Ferrara et al., 2013 [5]	Delphi consensus-based process involving a panel of Italian hematologists	Definition of patients not fit for intensive and non-intensive chemotherapy. The panel provides conceptual and operational criteria to evaluate the fitness of AML patients. These criteria are easily applicable in clinical practice for determining three fitness groups: fit, unfit, and frail.
Palmieri et al., 2019, 2020 [39]	Retrospective and real-life studies	- A cohort of 180 patients showed high concordance between fitness classes identified using the Ferrara criteria and overall survival. - In a retrospective study of 622 AML patients, the Ferrara criteria showed good accuracy in predicting 28-day and 100-day mortality. The authors concluded that the validity of the Ferrara criteria must be integrated with the molecular cytogenetic risk class of AML.
Borlenghi et al., 2018, 2021 [37]	Retrospective and real-life studies	- In a retrospective analysis of 208 patients >64 years with secondary AML, the authors integrated the Ferrara criteria with ELN risk classes. The Ferrara criteria correlated with the survival of fit, unfit, and frail subgroups. - The Ferrara criteria applied to 699 patients demonstrated the ability to predict survival. However, these criteria should be integrated with biological risk classes.
